# Molecular Approaches Reduce Saturates and Eliminate *trans* Fats in Food Oils

**DOI:** 10.3389/fpls.2022.908608

**Published:** 2022-06-02

**Authors:** James G. Wallis, Jesse D. Bengtsson, John Browse

**Affiliations:** Institute of Biological Chemistry, Washington State University, Pullman, WA, United States

**Keywords:** biotechnology, high-oleic, polyunsaturated fatty acid, oilseed, saturated fatty acid, *trans* fat, triacylglycerol, vegetable oil

## Abstract

Vegetable oils composed of triacylglycerols (TAG) are a major source of calories in human diets. However, the fatty acid compositions of these oils are not ideal for human nutrition and the needs of the food industry. Saturated fatty acids contribute to health problems, while polyunsaturated fatty acids (PUFA) can become rancid upon storage or processing. In this review, we first summarize the pathways of fatty acid metabolism and TAG synthesis and detail the problems with the oil compositions of major crops. Then we describe how transgenic expression of desaturases and downregulation of the plastid *FatB* thioesterase have provided the means to lower oil saturates. The traditional solution to PUFA rancidity uses industrial chemistry to reduce PUFA content by partial hydrogenation, but this results in the production of *trans* fats that are even more unhealthy than saturated fats. We detail the discoveries in the biochemistry and molecular genetics of oil synthesis that provided the knowledge and tools to lower oil PUFA content by blocking their synthesis during seed development. Finally, we describe the successes in breeding and biotechnology that are giving us new, high-oleic, low PUFA varieties of soybean, canola and other oilseed crops.

## Introduction

**Figure 1:**
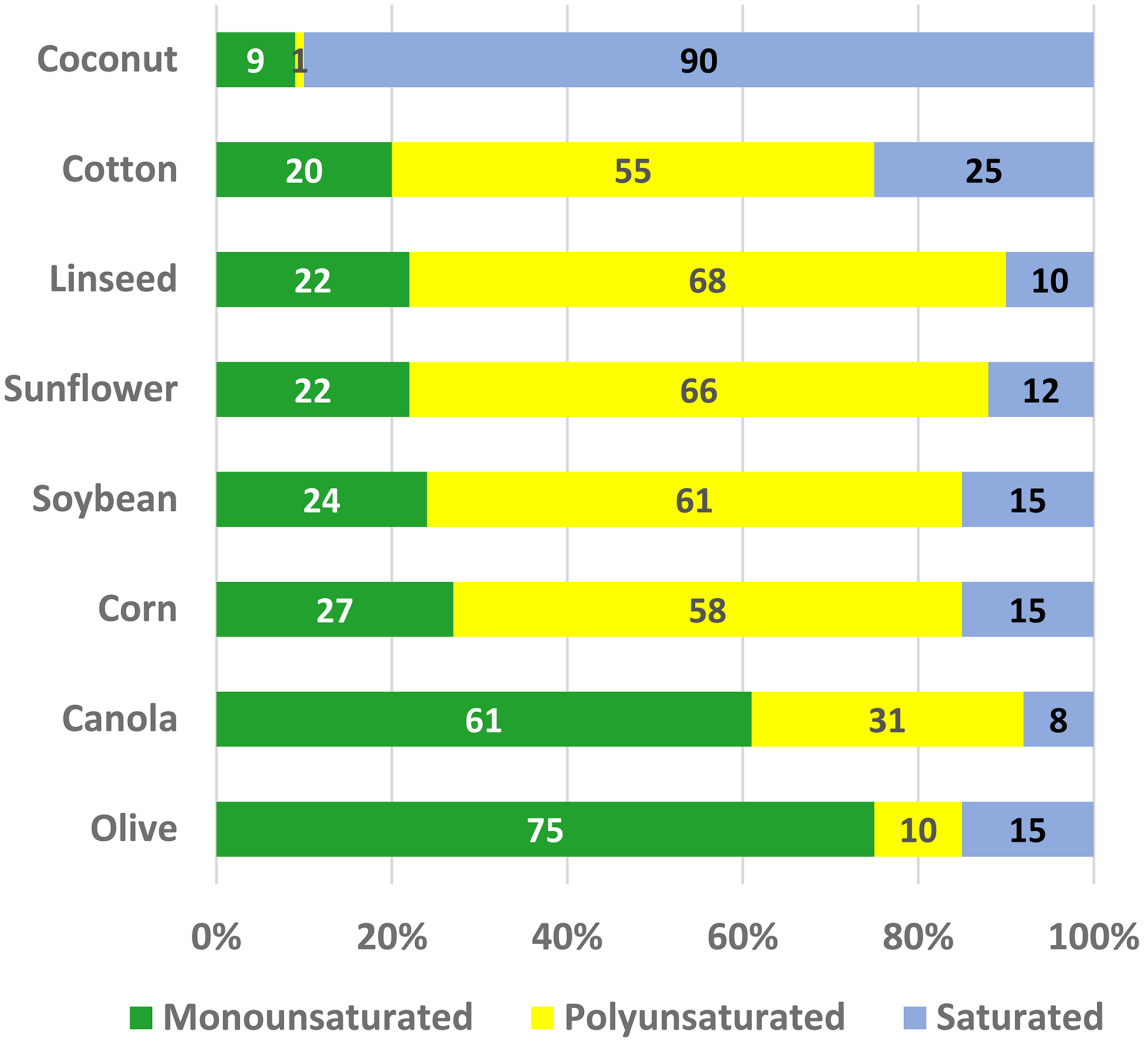
The oil compositions of some major crops. The percentages of monounsaturated (mainly 18:1), polyunsaturated (18:2 + 18:3), and saturated (16:0 + 18:0) fatty acids are shown.

Vegetable oils constitute one of the world’s most important plant commodities, with current annual production in excess of 605 million metric tons (USDA FAS, 2022) with a total value of $US244 billion (Kamble and Roshan, 2022). Consumption has increased steadily since 1970 at an average annual rate of 4%—about twice the rate of growth in world population. The major use of plant oils is in human and animal diets; in western diets plant oils and other fats contribute ~35% of calorie intake [CDC (US Centers for Disease Control), 2021]. Seed oils are composed almost entirely of triacylglycerols (TAG) in which fatty acids are esterified to each of the three hydroxyl groups of glycerol. The use of TAG as a seed reserve maximizes the quantity of stored energy for seedling germination and establishment, because the fatty acids are a highly reduced form of carbon (Graham, 2008; Baud and Lepiniec, 2010). A large variety of different fatty acid structures are found in nature (Hilditch and Williams, 1964; Gunstone, 1998), but just five account for >90% of the food oils produced: palmitic (16:0), stearic (18:0), oleic (18:1), linoleic (18:2), and α-linolenic (18:3) acids. These fatty acids are the ones also found most commonly in membrane lipids of plant cells (Ohlrogge and Browse, 1995). Monographs (Hilditch and Williams, 1964; Weiss, 1983) and searchable databases (Plantfadb.org; Ohlrogge et al., 2018) provide detailed information on oil compositions of many plant species. In Figure 1, we have summarized data from relevant oil crops to show the proportions of monounsaturated (mainly 18:1), polyunsaturated (PUFA; 18:2 plus 18:3) and saturated (16:0 and 18:0) fatty acids.

To a large extent the increases in oil production over the last 50 years have been fueled by the release of improved varieties and efficiencies of cultivation for a relatively few species—soybean (*Glycine max*), oil palm (*Elaeis guineensis*), canola (*Brassica napus*), and sunflower (*Helianthus annuus*). As a result, expansion of oil production has continued even though these major vegetable oils exhibit fatty acid compositions that make them less than ideal for human nutrition and the requirements of the food industry. For example, as shown in Figure 1, both traditional soybean and canola oils contain PUFA levels that threaten the shelf life of products made from them (Weiss, 1983; Gurr, 1992). Tropical oils, including palm and coconut, contain high levels of saturated fat, which are undesirable because they contribute to the development of atherosclerosis (Hooper et al., 2020). By contrast, monounsaturated oleic (18:1) and palmitoleic (16:1) acids protect against heart disease and metabolic syndrome (Gillingham et al., 2011). Modifying the fatty acid compositions of food oil crops is therefore an attractive goal that will permit diversification of agricultural production into a new generation of custom-designed crops (Msanne et al., 2020). As a result, there is unprecedented interest among plant biotechnology companies in modifying oil composition by changing the expression of endogenous genes, and by the use of cloned genes, to alter the products of seed lipid metabolism (Napier and Graham, 2010; Lu et al., 2011; Chen and Lin, 2013).

In this review, we shall first present the issues and challenges related to the fatty acid compositions of seed oils. Then we will describe how discoveries in the biochemistry, genetics, genomics and molecular biology of lipid metabolism have provided the tools and insight needed to engineer desirable changes in the fatty acid compositions of soybean, canola and other oilseed crops. These achievements have already led to the production of improved oilseed varieties.

## The Network of Pathways for Tag Synthesis in Oilseeds

**Figure 2:**
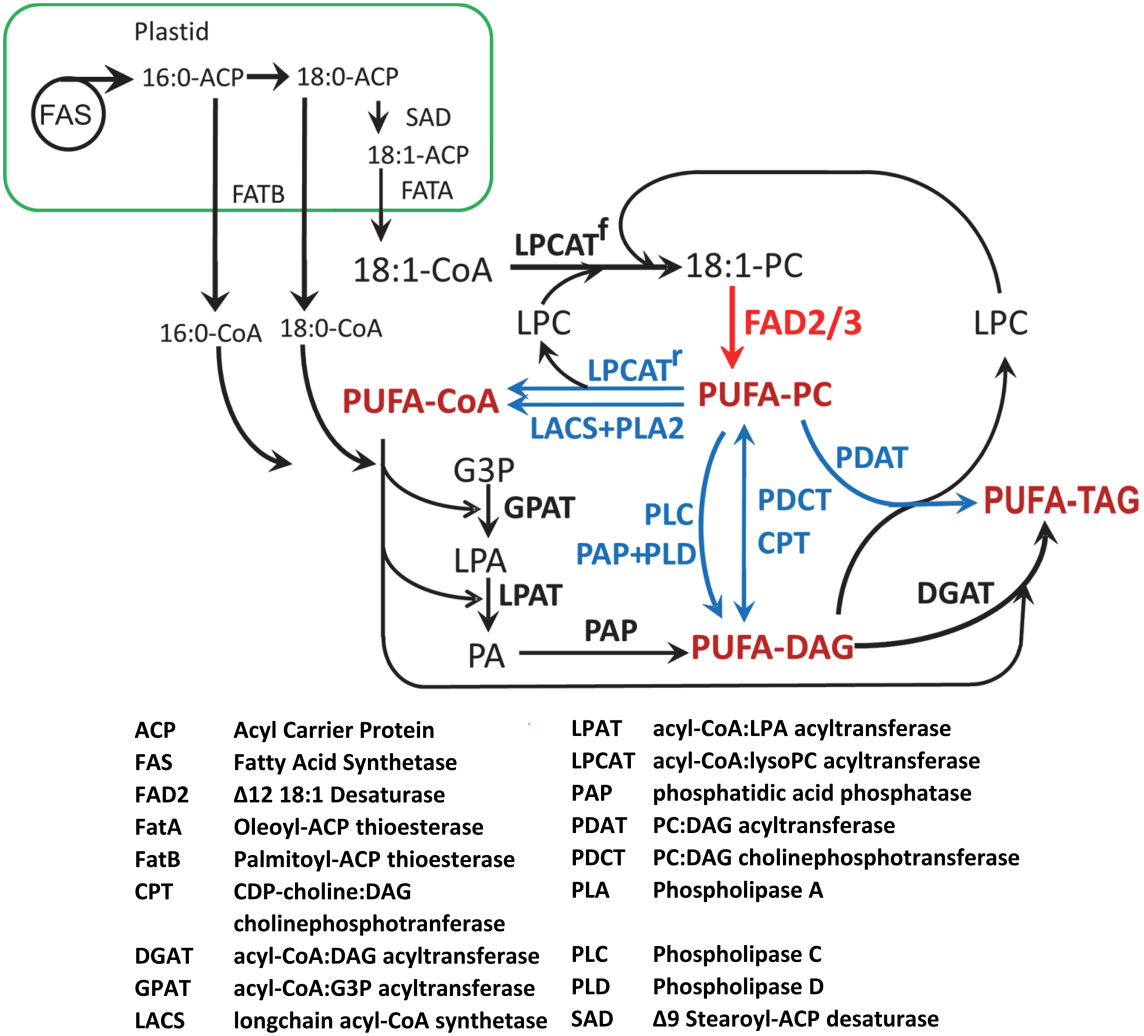
A simplified metabolic scheme for the synthesis of fatty acids and TAG in oilseeds. Following synthesis in the plastid, 16:0, 18:0, and 18:1 are exported to the cytoplasm and edoplasmic reticulum as acyl-CoAs. Saturated 16:0 and 18:0 are mainly incorporated through GPAT of the Kennedy pathway. Monounsaturated 18:1 may also enter the Kennedy pathway, but most is incorporated directly into PC by LPCAT. PUFA are synthesized from 18:1 on PC by the FAD2 desaturase, with some being further desaturated by FAD3 (red arrow). The three pathways that can mobilize PUFA from PC for TAG synthesis, shown in blue, are: (1) removal of PUFA from PC to the acyl-CoA pool by reverse LPCAT or combined PLA2 and LACS; (2) removal of the PC phosphocholine head group to produce PUFA-DAG by PDCT, reverse CPT, PLC, or PLD/PAP; (3) direct transfer to DAG by PDAT. Substrate abbreviations are as follows: DAG, diacylglycerol; G3P, glycerol-3-phosphate; LPA, lyso-phosphatidic acid; LPC, lyso-phosphatidylcholine; PA, phosphatidic acid; PC, phosphatidylcholine; TAG, triacylglycerol.

Most of the major oilseed crops (including soybean, canola, and sunflower) are characterized by oils containing predominantly 18-carbon unsaturated fatty acids plus lower proportions of saturated 16:0 and 18:0 that are also the major fatty acids of plant membrane lipids. The synthesis and accumulation of TAG in oilseeds occurs through a complex network of pathways located in the plastid, cytosol, endoplasmic reticulum, and lipid droplets. The pathways are summarized in Figure 2. Insights into the pathways and enzymes in this scheme have come from biochemical studies in many oilseeds (Voelker and Kinney, 2001; Bates et al., 2013) as well as genetic studies in the model oilseed Arabidopsis (Miquel and Browse, 1992; Browse et al., 1993; Lu et al., 2009; Wallis and Browse, 2010). The present formulation of the pathways shown in Figure 2 has benefited greatly from characterization of mutants (Lemieux et al., 1990; Miquel and Browse, 1992; Lu et al., 2009). Cloning of genes encoding several of the enzymes also depended on mutant analysis (Arondel et al., 1992; Okuley et al., 1994; Lu et al., 2009).

In plants, fatty acid synthase (FAS) is a multi-component (Type II) enzyme located in the plastid (Ohlrogge and Browse, 1995). The first committed step of fatty acid synthesis is the conversion of acetyl-CoA to malonyl-CoA by acetyl-CoA carboxylase (ACCase; Konishi and Sasaki, 1994). In dicots the ACCase is a multicomponent enzyme that is subject to feedback regulation by the actions of biotin attachment domain-containing (BADC) proteins (Salie et al., 2016; Keereetaweep et al., 2018). Following synthesis, the malonyl group of malonyl-CoA, is transferred to acyl carrier protein (ACP) by malonyl-CoA:ACP malonyltransferase (Lessire and Stumpe, 1983). The fatty acid synthase utilizes acetyl-CoA as the starting unit for condensation reactions, with malonyl-ACP providing two-carbon units in seven cycles of condensation and reduction to yield 16:0-ACP. Some 16:0 is released by thioesterases of the FATB family (Jones et al., 1995), but most is elongated through one more cycle of fatty acid synthesis to 18:0-ACP and efficiently desaturated by a stromal Δ9 stearoyl-ACP desaturase (Lindqvist et al., 1996) before being released by a FATA thioesterase. Thus, 16:0, 18:0 and 18:1 are the products exported from the plastid, after conversion to CoA thioesters by long-chain acyl-CoA synthetase (LACS) enzymes in the plastid envelope (Schnurr et al., 2002; Shockey et al., 2002), to become the primary substrates for glycerolipid synthesis in the endoplasmic reticulum.

The most direct route for TAG synthesis, sometimes called the Kennedy Pathway, could use only the 16:0-, 18:0- and 18:1-CoAs produced by the plastid to sequentially acylate glycerol-3-phosphate. The initial step in this pathway (Figure 2) is the transfer of an acyl group from acyl-CoA to the *sn*-1 position of glycerol-3-phosphate by acyl-CoA:glycerol-3-phosphate acyltransferase 9 (GPAT9) that generates lysophosphatidic acid (Shockey et al., 2016; Singer et al., 2016). Next, acyl-CoA:lysophosphatidic acid acyltransferase 2 (LPAT) forms phosphatidic acid (PA) by transferring an acyl group from the acyl-CoA to the *sn*-2 position of lysophosphatidic acid (Kim et al., 2005). Then, a phosphatidic acid phosphatase (PAP) enzyme removes the phosphate group at the *sn*-3 position to form diacylglycerol (DAG) before acyl-CoA:diacylglycerol acyltransferase (DGAT) adds a third acyl group to produce TAG. In this simple scenario TAG would contain only 16:0, 18:0, and 18:1 fatty acids, but seed oils also contain varying proportions of PUFA (Figure 1). In oilseeds conversion of 18:1 to 18:2 and 18:3 occurs on phosphatidylcholine (PC), the main structural lipid of the endoplasmic reticulum (Ohlrogge and Browse, 1995). In seeds, and also in other tissues, 18:1-PC is converted to 18:2-PC by fatty acid desaturase2 (FAD2), with some further desaturation to 18:3-PC by FAD3 (Figure 2, red arrow; Arondel et al., 1992; Okuley et al., 1994).

The transfer of 18:1 into PC for desaturation occurs by two main routes. The first and major route, acyl-editing (Bates et al., 2012), involves the transfer of 18:1 from 18:1-CoA to the *sn*-2 position of lyso-PC by acyl-CoA:lysoPC acyltransferase (LPCAT; Ståhl et al., 2004, 2008; Wang et al., 2012) and possibly other enzymes (Lager et al., 2015). The second is the conversion of 18:1-DAG to PC by either CDP-choline:DAG cholinephosphotransferase (CPT; Liu et al., 2015) or PC:DAG cholinephosphotransferase (PDCT; Lu et al., 2009; Figure 2).

Following the synthesis of PUFA on PC, there are three routes and at least seven possible mechanisms for the transfer of PUFA back into the pathways of TAG synthesis (Figure 2, blue). The LPCAT reaction is reversible, so that PUFA may enter the acyl-CoA pool and be used by the acyltransferases of the Kennedy pathway, and PUFA-CoA may also be formed by the combined actions of phospholipase A2 (PLA2) and LACS. The CPT reaction is also reversible, while the PDCT reaction is symmetrical, so that a new DAG molecule is generated each time 18:1-DAG is converted to PC. In principle, PUFA-PC may also be converted to PUFA-DAG by phospholipase C (PLC), or by phospholipase D (PLD) combined with PAP. Finally, direct transfer of the *sn*-2 fatty acid of PC to the *sn*-3 of DAG occurs by the action of phospholipid:DAG acyltransferase (PDAT; Dahlqvist et al., 2000; Mhaske et al., 2005). Lyso-PC is a co-product of the PDAT reaction and this can be reincorporated into the acyl editing cycle to channel 18:1 into PC for desaturation.

Metabolic labeling studies in various plant species has shown that the pathways and reactions within the network shown in Figure 2 contribute to TAG synthesis differently between species. In Arabidopsis, the DAG substrate for TAG formation comes mainly from PC (Bates and Browse, 2012), while acyl flux occurs primarily through the Kennedy pathway in other species. Once formed, nascent TAG accumulates between the leaflets of the endoplasmic reticulum bilayer membrane, which stimulates lipid droplet formation for sequestration in the maturing seed. New evidence indicates that, in some species, TAG molecules may be remodeled after synthesis by acyl-exchange reactions (Bhandari and Bates, 2021).

In the following sections, we will review the ways in which studies of lipid metabolism in Arabidopsis have resulted in new discoveries and a better understanding of the reactions, pathways and control of oil synthesis and accumulation in seeds, as well as new molecular tools that have allowed successful production of more-healthy oils.

## In Vegetable Oils Both Saturates and PUFA Present Challenges

### Saturated Fats Are Unhealthy

**Figure 3:**
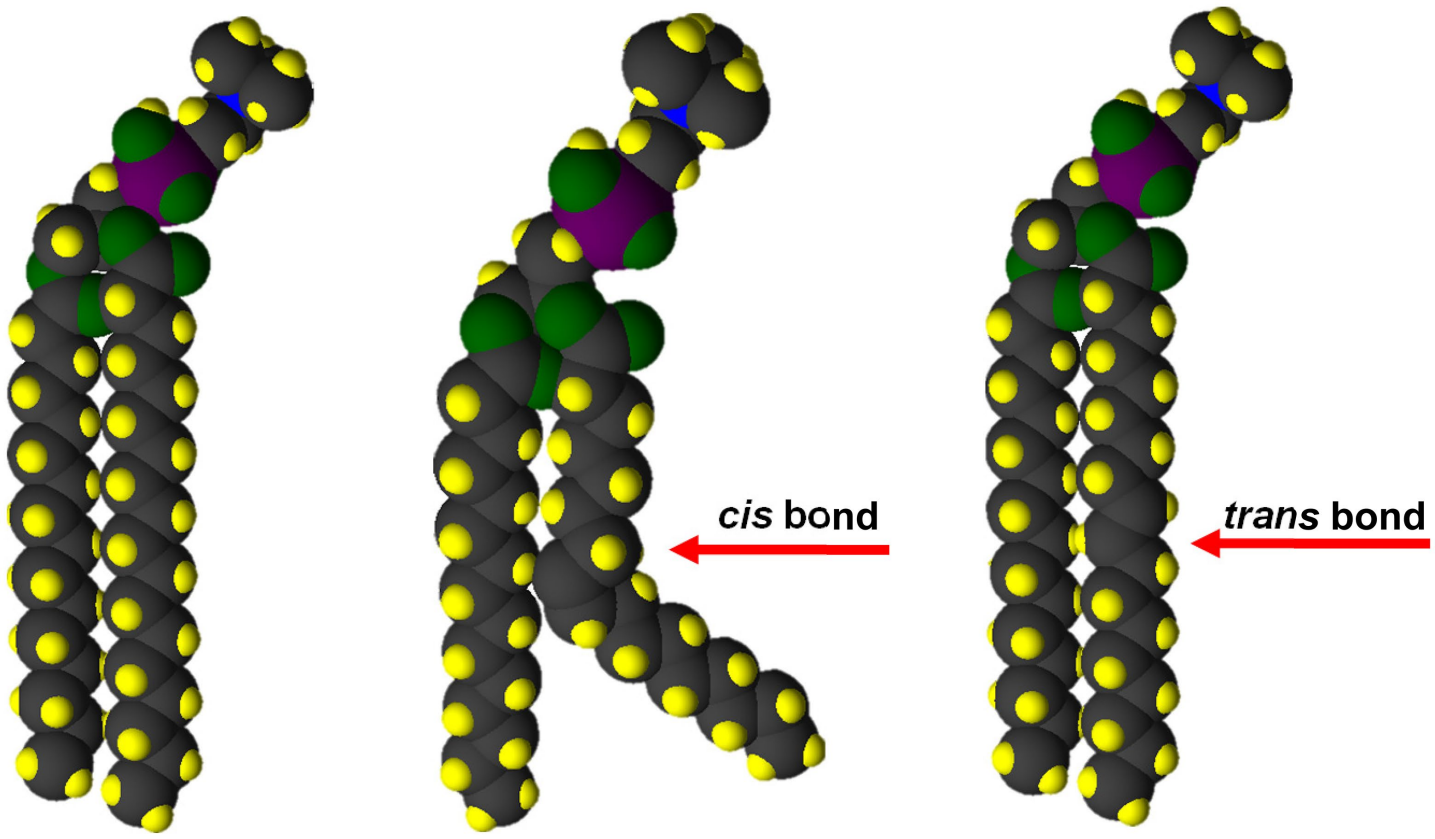
Only *cis* double bonds provide the membrane fluidity and functionality required for life. The bend in the fatty acid chain caused by a C=C *cis* double bond (center) disrupts Van der Waals interactions. Both saturated (left) and *trans* unsaturated (right) fatty acids provide for maximum Van der Waals interaction and a rigid structure.

Diets high in saturated fats are a known risk factor for heart attack, stroke and other diseases (Wang et al., 2016a,c). The reasons for this connection to health problems lie in the biophysical properties of saturated fatty acids. Animal fats (TAG), such as lard, beef tallow, and butter, contain 40%–70% saturates (Soyeurt et al., 2006), and are solid at room temperature because weak Van der Waals forces along the hydrocarbon chains of adjacent fatty acids favor the solid state (Kučerka et al., 2015). This property also encourages deposition of saturated TAG and fatty acid in atherosclerotic plaques in blood vessels, raising the risk for heart attack and stroke. The presence of a single, *cis* double bond produces a bend in the fatty acid chain that prevents extensive Van der Waals interactions (Figure 3). As a result, the melting temperature of lipids containing monounsaturated fatty acids are close to 0°C compared with ~50°C for saturated lipids (Dratz and Deese, 1986; Niebylski and Salem, 1994; Stillwell and Wassall, 2003; Lin et al., 2017).

Although the biophysical issues are more complicated in biological membranes, the same properties of saturated fatty acid also make them unsuitable, by themselves, as components of the glycerolipids that make up the bilayer of these membranes. The membranes of essentially all cellular organisms require the majority of fatty acid in their glycerolipids to be *cis*-unsaturated to provide the fluidity and function to support cell processes (Pilch et al., 1980; McConn and Browse, 1998; Ballweg and Ernst, 2017).

### Polyunsaturated Fatty Acids Become Rancid by Oxidation

In plants, the predominant PUFA are 18:2 and 18:3, which are major components of many seed oils (Figure 1). These same PUFA perform essential functions as constituents of plant membranes and as substrates for plant hormone synthesis. As constituents of membrane lipids, PUFA are vital to a wide range of membrane functions, conferring fluidity, flexibility, and selective permeability to membranes (Los et al., 2013), while also supporting efficient photosynthesis (Allakhverdiev et al., 2009). These PUFA affect many other metabolic and physiological processes, including cold adaptation and survival (Liu et al., 2013; Hou et al., 2016), heat adaptation (Falcone et al., 2004), modulation of ion channels (Gutla et al., 2012; Elinder and Liin, 2017), as well as endocytosis/exocytosis (Murphy et al., 2005; König et al., 2008; Doherty and McMahon, 2009). PUFA have roles in optimizing the activities of membrane-associated enzymes that are sensitive to biophysical properties of lipid membranes (Upchurch, 2008). The oxylipin jasmonic acid is derived from 18:3 and, as the hormone jasmonyl-isoleucine, helps regulate insect and pathogen defense, reproductive development, and other key processes (Browse, 2009; Chung et al., 2009; Browse and Wallis, 2019).

PUFA play similarly important structural and signaling roles in animals, but mammals have no orthologue of the plant FAD2 desaturase (Figure 2), and thus are unable to synthesize PUFA *de novo* (Wallis et al., 2002), so suitable fatty acids must be acquired in the diet. Mammals require both 18:2 and 18:3 as essential fatty acids, and these are used as substrates to synthesize longer chain fatty acids, 20:4 n-6 (arachidonic acid), 20:5 n-3 (eicosapentaenoic acid) and 22:6 n-3 (docosahexaenoic acid). These very-long-chain PUFA are vital membrane components, especially in the brain and nerve tissues (Kaur et al., 2014). They are also substrates for cyclooxygenase enzymes in pathways that lead to the synthesis of the n-3 and n-6 families of eicosanoids, including prostaglandins, leukotrienes, and thromboxanes, that bind to specific G-protein-coupled receptors and signal cellular responses that mediate fever, inflammation, vasodilation, blood pressure, and pain (Saini and Keum, 2018).

Meat is a relatively poor source of essential fatty acids (Saini and Keum, 2018). Vegetable oils are the major source 18:2 and 18:3 in human diets; however, it has been calculated that the minimum required intake of these PUFA is typically met from other plant sources, such as green leafy vegetables (Johnson et al., 2018). Furthermore, because of the inefficiencies of human PUFA metabolism it is actually preferable to obtain very-long-chain PUFA directly from eating fish and other marine animals (Mori, 2014). Extracted fish oils are also widely used as dietary supplements. The origin of very-long-chain PUFA, 20:4 n-6, 20:5 n-3, and 22:6 n-3, found in fish is microalgae (phytoplankton); the PUFA synthesized in algae are concentrated by the food chain into fish tissues (Ryckebosch et al., 2012). Several microalgae have also been grown in culture as sources of PUFA (Vrenna et al., 2021). For these reasons, the PUFA components of plant oils are not required for optimum nutrition in humans and other mammals. The fact that PUFA are inessential components of plant oils is fortunate because, as described below, their presence has been a challenge to food processing for more than a century—and for storage of oils for millennia.

The dietary requirement for PUFA is complicated by differences in human metabolism that result from consuming n-3 and n-6 fatty acids. Both n-3 and n-6 very-long-chain PUFA are metabolized to produce eicosanoid regulatory molecules, but the derivatives of the n-3 fatty acids, 20:5 and 22:6 quell excitation of the inflammatory response in tissues, while an excess of 20:4 n-6 derivatives have the opposite effect, elevating inflammation in a variety of tissues (Serhan and Savill, 2005). Persistent inappropriate stimulation of the inflammatory response is linked to development of cardiovascular disease (Harris et al., 2007) and cancer (Kwon, 2016). Human health is damaged when intake of n-6 fatty acids greatly exceeds that of n-3 fatty acids. In contrast, consumption of high levels of n-3 fatty acids are correlated with reductions in diabetes, obesity, osteoporosis, and some forms of neurological degeneration (Natto et al., 2019). The key to health is in the balance of n-3 and n-6 fatty acids (Simopoulos, 2003). The high levels of the n-6 PUFA, 18:2, from vegetable oils in the diets of many countries has led to efforts to enrich diets for n-3 fatty acids by promoting changes in diet to fish oils or designing food-stuffs that are n-3 enriched. In this respect, green leafy vegetables, which contain a high ratio of 18:3 n-3 to 18:2 n-6, are a good source of these essential fatty acids. Some seed and nut oils also offer naturally high levels of n-3 fatty acids (Kuhnt et al., 2012). Successful transgenic modification of commercial oil crops to produce very-long-chain PUFA have recently obtained regulatory approval in some countries, and may become important sources of these fatty acids, particularly by providing plant-based feed stocks for aquaculture (Napier et al., 2019; Han et al., 2020).

**Figure 4:**
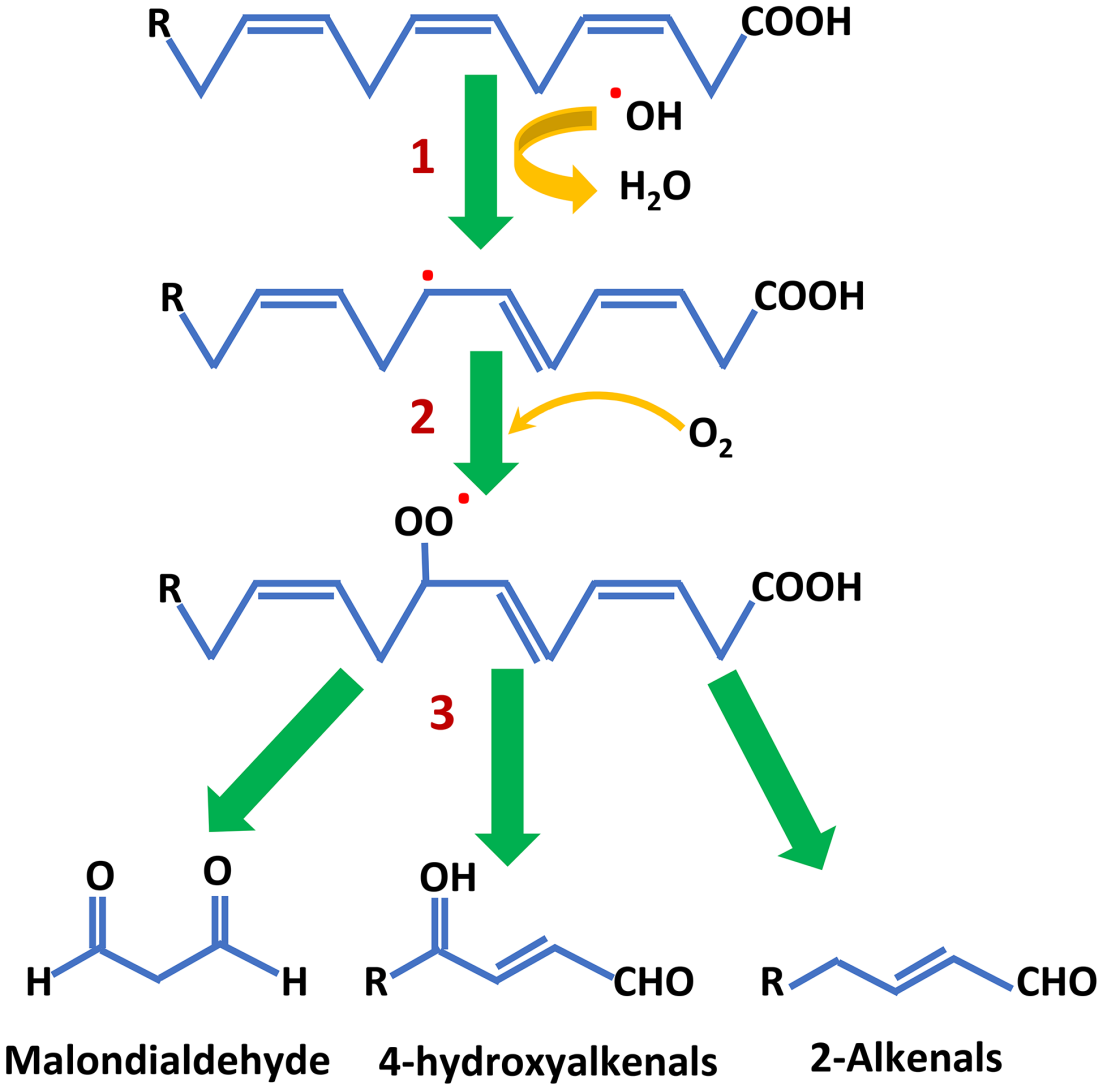
An abbreviated scheme of PUFA oxidation that results in rancidity. 1. Reaction of the *cis*, *cis*-1,4-pentadiene structure with reactive oxygen species (shown here as the hydroxyl radical) produces a PUFA free radical. 2. Addition of molecular oxygen produces a peroxy radical. 3. Intramolecular rearrangements and further oxidation leads to chain cleavage and the production of a number of low molecular weight products. Note that the peroxy radical can abstract a proton from another PUFA molecule to propagate and accelerate PUFA breakdown.

A pervasive risk from PUFA chemistry is the propensity of these fatty acids to undergo oxidation. The susceptibility to oxidation increases considerably with each additional double bond in the carbon chain. In both plants and animals, membrane integrity and function are threatened by oxidation initiated by reactive oxygen species generated during photosynthesis, respiration and other processes (Foyer and Shigeoka, 2011; Smirnoff and Arnaud, 2019). Living cells employ a range of metabolic defenses to limit and repair this damage (Gill and Tuteja, 2010; Das and Roychoudhury, 2014; Zandalinas et al., 2020). For vegetable oils—and fish oils—PUFA oxidation during storage and food manufacture are a particular challenge, especially during high-temperature applications. Free radical formation by abstraction of a hydrogen atom and addition of molecular oxygen to the *cis*, *cis*-1,4-pentadiene structures of PUFA leads to the production of aldehydes, alcohols, and fatty acid peroxides (Figure 4; Schneider, 2009). Isoprostanes are also formed and these are a health concern because they can mimic prostaglandins (Roberts and Milne, 2009). The rancid smell of oxidized oils during storage, or in baked goods and other food products, comes from release of these compounds as PUFA break down. The process is greatly accelerated when oils containing PUFA are heated, for example in frying. The addition of antioxidants and oil blending can ameliorate the effects of oxidation to some extent (Mishra et al., 2021) but, as described below, conversion of PUFA by partial hydrogenation has previously been widely used to stabilize oil and produce margarine and other solid fats.

## Strategies to Lower Saturate Content of Vegetable Oils

### Desaturase Genes

Some plant TAGs contain high proportions of saturated fatty acids that are comparable to levels in animal fats. For example, cocoa butter (*Theobroma cocao*) contains 60% 16:0 + 18:0 (Lipp et al., 2001; Gilabert-Escrivá et al., 2002), while palm oil has 50% saturates (Mukherjee and Mitra, 2009). The same health issues are a concern with these in food products. Even though most food oils have much lower proportions of saturates (Figure 1), it is nevertheless desirable to reduce the levels further. One strategy that has been investigated for this purpose is to desaturate 16:0, which is the major saturated fatty acid in most plant oils.

Macadamia nuts (*Macadamia integrifolia*) and seeds of the subtropical cat’s claw vine (*Doxantha unguis-cati*) have variant acyl-ACP desaturases (Cahoon et al., 1998) that convert a major proportion of 16:0 to 16:1 Δ9 (palmitoleate). To investigate whether the cat’s claw enzyme could reduce the level of saturates in plant oils, Bondaruk et al. (2007) cloned a cDNA encoding this enzyme and expressed it in developing seeds of Arabidopsis and canola. Although the transgenic desaturase catalyzed synthesis of 16:1 (some of which was elongated to longer-chain monounsaturates), the proportion of 16:0 in the seed oil was reduced only slightly—14% in Arabidopsis and less than 5% in canola (Bondaruk et al., 2007). In another study, site-directed mutagenesis of the castor (*Ricinus communis*) 18:0-ACP desaturase based on analysis of the crystal structure of the protein (Lindqvist et al., 1996) produced a modified enzyme (COM25) with high activity and specificity towards 16:0 (Whittle and Shanklin, 2001). Seed from transgenic plants expressing this engineered desaturase contained large increases in 16:1 and its elongation products, but no significant decrease in 16:0, or in total saturates in the oil (Cahoon and Shanklin, 2000; Nguyen et al., 2010). Expression of the COM25 desaturase in mutants of lipid metabolism, such as *fab1* (16:0-ACP elongase, KASII), or *fae1* (acyl-CoA elongase), resulted in larger increases in 16:1, but also increased 16:0—to 19%–22% compared to 10% in the non-transgenic controls (Nguyen et al., 2010). These results indicate that targeting 16:0-ACP for desaturation immediately following its synthesis on the plastid fatty acid synthase is not a successful strategy for reducing saturate content of seed oils.

More fruitful oilseed engineering efforts have focused on desaturating fatty acids following export from the plastid. Several of these experiments used animal acyl-CoA desaturases expressed in seed tissue. A stearoyl-CoA desaturase from rat was expressed in soybean seeds with a seed-specific promoter. This enzyme acts on fatty acids as acyl-CoAs, rather than acting on acyl-ACPs. Compared to controls, transgenic lines with the rat desaturase expressing seeds had 0.6% less 16:0, and <0.1% less 18:0 (Moon et al., 2000). A more successful experiment used a *Caenorhabditis elegans* acyl-CoA desaturase specific to 16:0 (Watts and Browse, 2000). This desaturase, FAT5, when expressed in Arabidopsis seeds, reduced saturated fatty acid content by 65%, from 8.6% to 3.0% of total fatty acids in the oil (Fahy et al., 2013). In vegetative tissues, 16:0 is essential for proper growth and development, and large reductions in 16:0 content resulted in poor germination, establishment, and defective growth (Bonaventure et al., 2003). However, seed-specific FAT5 expression did not result in compromised seed characteristics, or plant development (Fahy et al., 2013).

Other strategies to reduce saturated fatty acid content in seed oil have used cyanobacterial glycolipid desaturase. Cyanobacteria desaturases use ferredoxin as an electron carrier rather than cytochrome b5 used in eukaryotic desaturases. A desaturase from *Synechococcus elongatus* has activity when targeted to the leaf chloroplasts (Ishizaki-Nishizawa et al., 1996), but its utility in oil seed engineering seemed limited (Bai et al., 2016). However, by adding an endoplasmic reticulum targeting sequence, and subjecting the gene to random mutagenesis in the desaturase deficient yeast (*ole1Δ*), variants with high levels of activity in eukaryotic systems were identified, and when these variants were expressed in Arabidopsis seed, initial transformants had reduced 16:0 levels, comparable to the best homozygous FAT5 lines, but also had reductions in 18:0, unlike the 16-carbon specific FAT5 (Bai et al., 2016).

### Blocking 16:0 Export From the Plastid

Expression of desaturases in the endoplasmic reticulum has been successful in reducing saturates by 65% in Arabidopsis seed oil. Strategies targeting the enzyme that allows for export of saturated fatty acids from the chloroplast, fatty acid thioesterase B (FatB) have also yielded seeds with low saturated fatty acid content. Analysis of *fatb* mutants in Arabidopsis showed that knockout of the gene results in 3.6% 16:0 in seed oil compared to 8.6% in controls. However, the *fatb* knockout mutation severely compromised growth, with deleterious alterations throughout lipid metabolism, including defective sphingolipid and chloroplast lipid biosynthesis (Bonaventure et al., 2003). These results indicate that molecular techniques, such as CRISPR/Cas9, that produce constitutive knock out of the *FATB* gene are not viable as a means to reducing saturates in seed oils. For this reason, a number of engineered oilseeds with low saturated and high monounsaturated fatty acids content are based on seed-specific reductions in *FATB* expression (Ozseyhan et al., 2018; Wood et al., 2018). As described later, the Vistive Gold soybean produced by Monsanto (now Bayer), harbors a seed-specific RNAi construct that reduces *FATB* expression.

## Industrial Chemistry Is the Wrong Solution to the PUFA Problem

Beginning early in the twentieth century, food processors addressed the problem of PUFA oxidation and rancidity by subjecting vegetable oils to a process of partial hydrogenation (Korver and Katan, 2006). The process of hydrogenation involves heating the oil in a closed (oxygen free) reaction vessel in the presence of hydrogen gas and a nickel-based catalyst. Under these conditions, hydrogen is added across the double bonds to generate saturated C-C single bonds. The reaction may be continued to completion to generate a product with fully-saturated, solid TAG; however, in most applications, the process is stopped in order to yield products that range from lightly hydrogenated oils used for cooking or as salad oils, to more highly hydrogenated semi-solid fats such as margarine. These partially hydrogenated fats have been used in baked goods, commercial food preparation and in margarines for many years. However, during partial hydrogenation, many of the *cis* double bonds present in the original oil are converted to *trans* isomers. As indicated earlier, *cis*-unsaturated fatty acids are essential for life on earth, because the presence of *cis* double bonds in a fatty acid esterified to glycerolipids produces a bend in the fatty acid chain that reduces van der Waals interactions and provides the molecular mobility and fluidity required to maintain the structure and biological function of membranes of living cells. All-*trans* fatty acids behave biophysically, and thus physiologically, like saturated fatty acids (Figure 3). Furthermore, *trans* fatty acids are poorly metabolized by humans, leading to their accumulation, along with cholesterol, in tissues and blood vessels (Mozaffarian et al., 2006; Micha and Mozaffarian, 2008, 2009; Wang et al., 2016c).

Only very low amounts of *trans* fatty acids appear naturally in foods. Industrial production of *trans*-enriched oils greatly increased the consumption of these formerly rare fatty acids. Industrial production of *trans* fats developed rapidly from its inception early in the last century (Korver and Katan, 2006) and they were widely used to replace saturated fats, such as butter and lard, that were known to be unhealthy. Typically, in foods containing partially hydrogenated oils, *trans* fats made up 10%–30% of the total fat content (Satchithanandam et al., 2004). However, health concerns about *trans* fats themselves were raised when research revealed that their consumption increased blood cholesterol levels (Mensink and Katan, 1990; Troisi et al., 1992). By the mid-1990’s alarms were sounded about the connections between *trans* fatty acids and risk factors for coronary heart disease, including atherosclerosis, inflammation and calcification of arteries (Willett et al., 1993; Willett and Ascherio, 1994; Ascherio et al., 1999; Mozaffarian et al., 2006), as well inhibition of prostacyclin synthesis required for blood flow regulation (Kummerow et al., 2013). In some countries, *trans* fat consumption exceeded 7% of total fat consumed beginning in the mid-1970s; consumption in North America was particularly high (Craig-Schmidt, 2006; Micha et al., 2014). Consumption of *trans* fats was shown to have additional unforeseen negative effects on human health, including increased risk of diabetes, cancer, and inflammatory diseases (Kiage et al., 2013; De Souza et al., 2015; Dawczynski and Lorkowski, 2016; Kwon, 2016).

The dangers of foods containing *trans* fats were well established by 1998, but objections and prevarication from industry interests, as well as lethargic responses from some regulatory agencies delayed elimination of *trans* fats from foods for 20 years. Worldwide, more than half a million deaths per year have been attributed to high levels of *trans* fat consumption (Wang et al., 2016a), suggesting a 20-year global death toll of 10 million people. In the U.S., the 20,000 excess deaths in 2010 conservatively estimated by the Centers for Disease Control as being due to *trans* fats is equivalent to deaths from opioid overdoses in that year [NIDA (National Institute on Drug Abuse), 2020].

## FAD2 Is the Gateway to PUFA Synthesis

**Figure 5:**
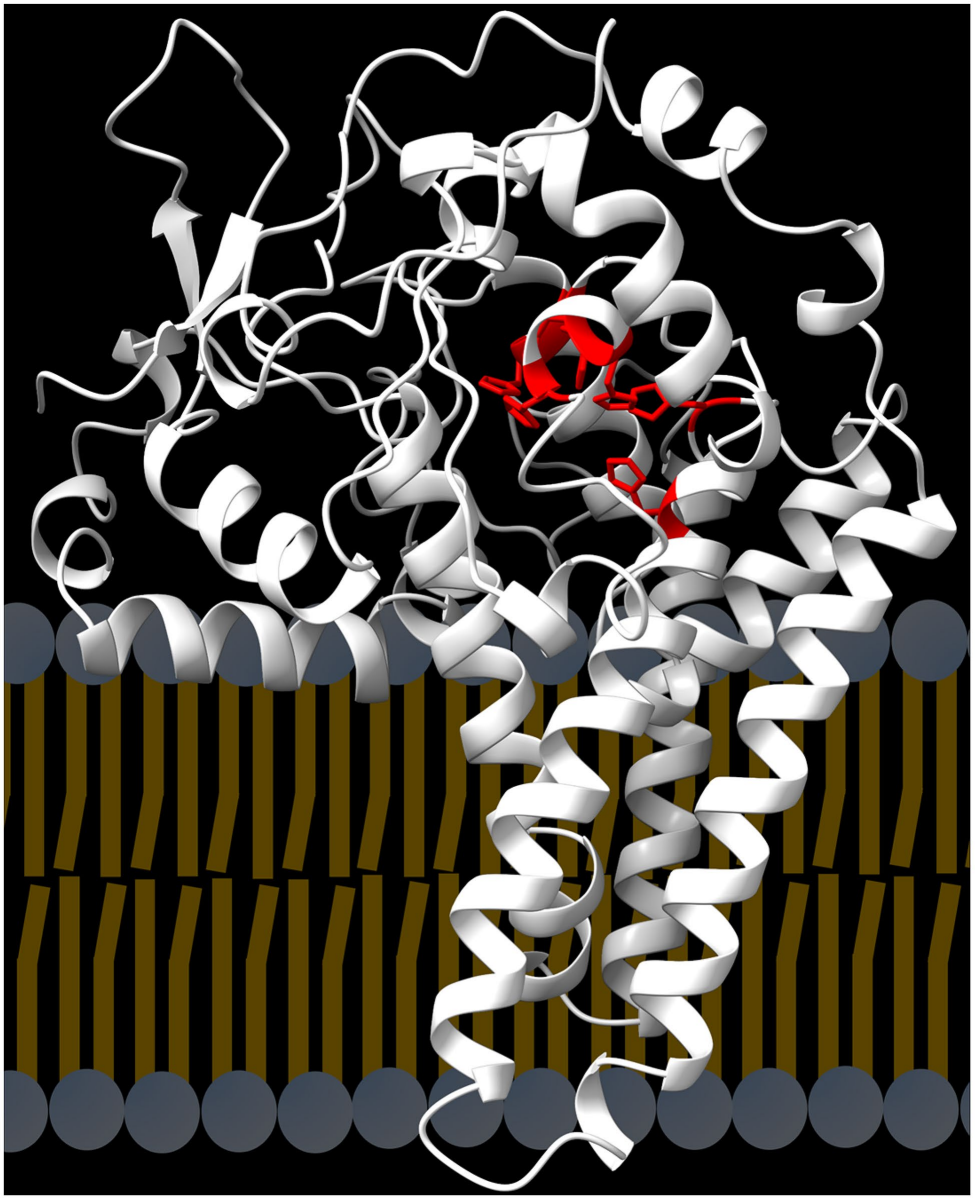
Structure of the FAD2 desaturase. The protein contains four hydrophobic, α−helices that span the enodplasmic reticulum bilayer. In the folded protein the three conserved histidine boxes (red) are brought together to coordinate the diiron-oxo complex at the active site.

Synthesis of the PUFA 18:2 from 18:1 is catalyzed by a desaturase (FAD2) that acts on 18:1 esterified to PC, the major structural glycerolipid of the endoplasmic reticulum (Figure 2). Analysis of the FAD2 amino-acid sequence identified four hydrophobic, α-helical segments predicted to span the bilayer membrane and three conserved histidine boxes that coordinate the oxo-bridged diiron complex at the active site (Okuley et al., 1994). The Alpha-fold model of the Arabidopsis FAD2 protein (Figure 5) shows how the four hydrophobic α-helical segments are predicted to anchor the protein into the endoplasmic reticulum, while an additional amphipathic α-helix is shown at the membrane-cytoplasm interface (Goddard et al., 2018; Jumper et al., 2021; Pettersen et al., 2021; Varadi et al., 2021). In the folded protein, the three histidine-boxes are brought together to form the active site within the protein domain that sits outside the plane of the membrane.

Following identification of the plant 18:1-PC desaturase (Stymne and Appelqvist, 1978), biochemical and genetic analyses of Arabidopsis *fad2* mutants (Miquel and Browse, 1992, 1994; Miquel et al., 1993) provided a detailed understanding of the biochemical role of the 18:1-PC desaturase in membrane biology and seed-oil synthesis. The FAD2 enzyme is responsible for ~95% of PUFA synthesis in Arabidopsis and other oilseeds (Voelker and Kinney, 2001; Bates et al., 2013). In the null *fad2-2* mutant of Arabidopsis, PUFA are reduced to 3% of seed fatty acids. The remaining fraction is produced by the plastid FAD6 enzyme (Browse et al., 1989). For this reason, reducing or eliminating FAD2 expression is an attractive strategy for greatly lowering the PUFA content of soybean, canola, and other plant oils. In principle, blocking transfer of 18:1 into PC (Figure 2) would also reduce PUFA synthesis; however, combining mutations in *ROD1* (PDCT), *LPCAT1* and *LPCAT2* in Arabidopsis reduced PUFA by only 65% (Bates et al., 2012) because the essential CPT activity catalyzes *de novo* synthesis of PC (Figure 2). The cloning of the Arabidopsis *FAD2* gene (At3g12120) by T-DNA tagging (Okuley et al., 1994) allowed the identification and cloning of *FAD2* genes from many plant species, including soybean and canola.

Arabidopsis contains a single *FAD2* gene that is responsible for 18:1 desaturation in all tissues. Plants containing the null *fad2-2* mutation are damaged and killed by low temperatures (Miquel et al., 1993) and *fad2-2* seeds are compromised in development and germination (Miquel and Browse, 1994). These results indicated that strategies to reduce FAD2 activity and PUFA content of oilseeds need to be carefully designed and investigated. Early success in reducing PUFA in soybean was possible because cloning experiments using Arabidopsis *FAD2* as a probe identified two classes of *FAD2* genes. *GmFAD2-2* encodes a constitutive enzyme, while two *GmFAD2-1* genes (*GmFAD2-1A and FAD2-1B*) are expressed only in developing seeds (Heppard et al., 1996; Kinney, 1996). By using a transgene construct to suppress expression of the two seed specific genes, scientists at Dupont Co. successfully produced lines whose seed oil contained <10% PUFA. These lines were available by the mid-1990s, and could have been used to replace partially hydrogenated soybean oil, thereby reducing dietary intake of *trans* fats, providing an attendant reduction in the burden on human health and wellbeing. Unfortunately, the agricultural and food industries were not interested in considering the new oils because partial hydrogenation of conventional soybean oil was cheap, and because the new cultivars were based on transgenic technology. High-oleic varieties of canola and sunflower were also available in the 1990s (Schuppert et al., 2006; Tarrago-Trani et al., 2006), but these were limited to specialty markets because the food industry, especially in the United States, remained wedded to hydrogenation as a means to stabilize vegetable oils and to produce spreads and shortenings.

## Committing to Eliminating *trans* Fats

The accumulating evidence, for example from the Nurses’ Health Study at Harvard, documenting the detrimental effects of *trans* fats on human health (Oh et al., 2005; Ardisson Korat et al., 2014) led to calls from health advocates in the US and other countries to restrict *trans* fats in food. The rate of reduction and elimination of *trans* fats from food has varied greatly from country to country. As early as 1995 industry in the Netherlands and other European countries began voluntary reductions of levels by reformulation of margarines (Katan, 2006). In 2003 voluntary labeling began in Denmark (Leth et al., 2006). In these and other European countries, this early appreciation of dangers of *trans* fats in food led to reformulation of many food products to reduce *trans* fats (List, 2014; Wang et al., 2016b). One method to achieve this was to carry out hydrogenation to completion to provide a fully saturated TAG, then mixing this with untreated oil and using lipase enzymes to bring about interesterification of the TAG fractions to achieve a desired semi-soft or hard product (Sivakanthan and Madhujith, 2020). As a result, *trans*-fat intake in Western Europe in the years 1990–2010 was 60% less than that in North America (1.1% vs. 2.9% of energy; Micha et al., 2014), even though U.S. margarine producers made some efforts to reformulate their products during this period (Tarrago-Trani et al., 2006).

The Canadian government required mandatory labeling of *trans* fat content on foods beginning in the 2005. Concerns expressed to the United States Food and Drug Administration (FDA), beginning in 2000 took some time to persuade regulators. The FDA eventually ruled that after 1 January 2006 the level of *trans* fat must be specified on all food products. The inclusion of this new line item in Nutrition Facts labels greatly boosted public awareness of the dangers of *trans* fats and was followed in the same year by New York City limiting the use of *trans* fats in restaurants (L'abbé et al., 2009). Publicity about these efforts drove consumer opposition to partially hydrogenated oils and resulted in rapid declines in *trans*-fat consumption. This led to reduced demand for soybean oil in favor of canola oil and other alternatives (Goldsmith, 2008). The result was a 32% drop in market share for soybean oil between 2006 and 2014 (United Soybean Board, 2013). Finally, prompted in part by a petition and subsequent litigation (Amico et al., 2021), the FDA agreed in 2015 to ban *trans* fats from foods with final implementation in 2018.

Elsewhere in the world, labeling, limits on *trans*-fat content, and some outright bans on *trans* fats in food were instituted in many countries (Restrepo and Rieger, 2016; Downs et al., 2017). These actions produced rapid declines in global *trans*-fat consumption, although several countries still use more than the World Health Organization (WHO) benchmark for safe consumption (Wanders et al., 2017). The WHO has an active program to convince countries to lower *trans* fatty use [WHO (World Health Organization), 2019].

## Improved Oils by Design

**Figure 6:**
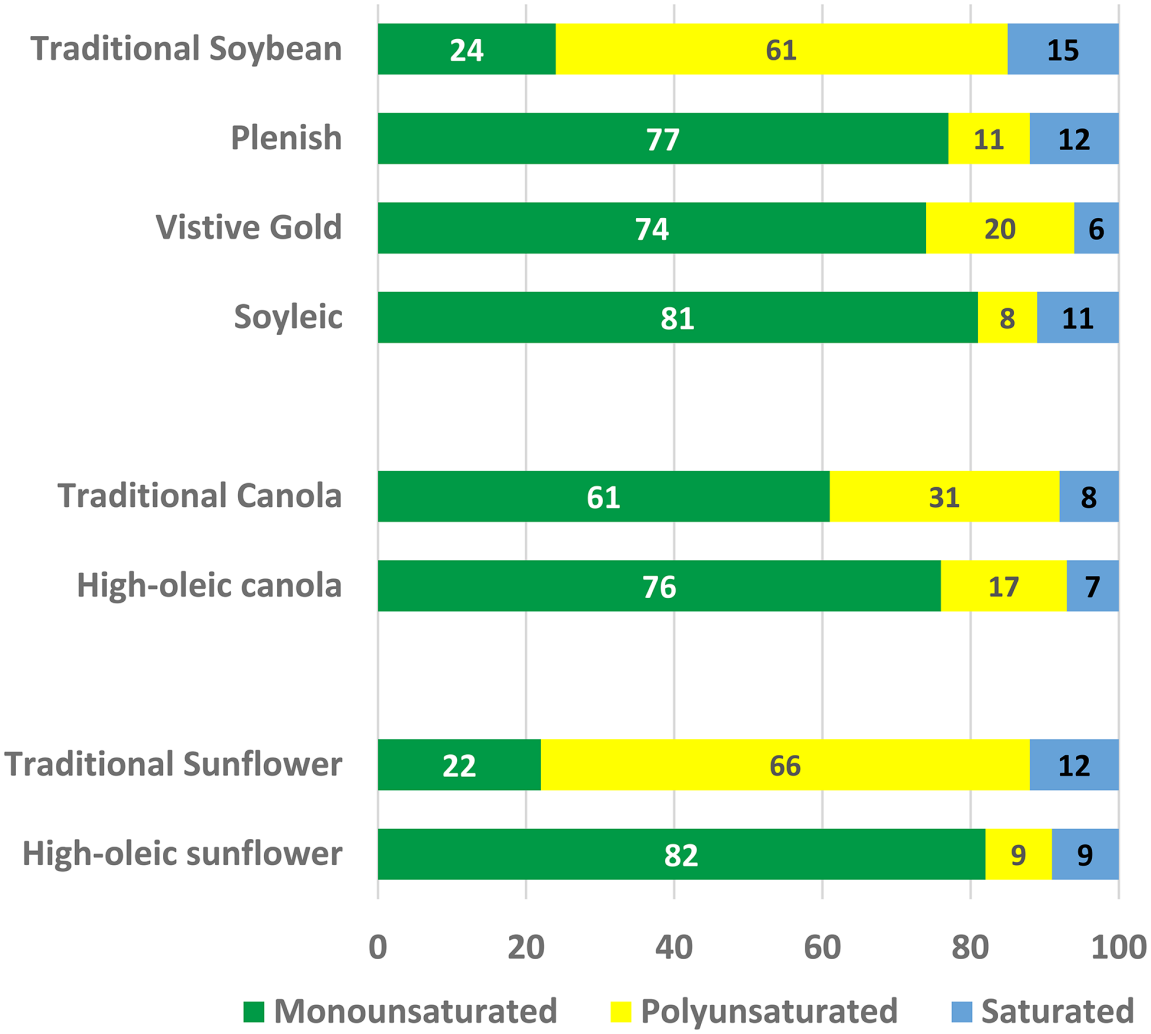
The oil compositions of high-oleic varieties of soybean, canola, and sunflower. The percentages of monounsaturated (mainly 18:1), polyunsaturated (18:2 + 18:3), and saturated (16:0 + 18:0) fatty acids are shown.

The cloning and characterization of the Arabidopsis *FAD2* gene was the key to understanding the genetics of high-oleic oilseed varieties in crops species such as canola and sunflower (Dar et al., 2017), and to the development of strategies that quickly led to the production of high-oleic soybean through gene silencing of the two seed-specific *FAD2* homologues, *GmFAD2-1A* and *GmFAD2-1B* (Heppard et al., 1996). As discussed earlier the resulting varieties were not initially accepted by the wider food industry. The high-oleic line developed by DuPont Co. (now Corteva Inc.), Plenish, has a doubly attractive fatty acid profile because the down regulation of *FAD2* genes not only reduces PUFA in favor of monounsaturated 18:1, but also leads to a reduction in 16:0 content to 12%, compared to 15% in regular soybean oil (Figure 6; Heppard et al., 1996). The approach taken by Monsanto (now Bayer) scientists, in addition to targeting the *FAD2-1* genes, included the seed-specific suppression of the *GmFatB* gene. This additional gene knockdown results in a substantial additional decline in 16:0 to 6%, thereby further improving the fatty acid profile of this soybean oil marketed as Vistive Gold (Figure 6). For soybean and other crops with seeds-specific *FAD2* genes other genomic techniques of gene silencing, such as TALENs and CRISPR/Cas9 are also appropriate and have been used to reduce soybean oil PUFA content (Haun et al., 2014; Demorest et al., 2016).

The fact that the *GmFAD2-1* genes show seed-specific expression indicates that traditional mutation breeding should provide comparable changes in oil composition without requiring transgenic approaches. Indeed, the identification of mutations in both *GmFAD2-1A* and *GmFAD2-1B* genes and their combination produced a soybean line with 18:1 and PUFA levels (Figure 6) comparable to those in Plenish soybean oil (Pham et al., 2010, 2011; Shi et al., 2015), and derived varieties are now marketed by the Missouri Soybean Merchandising Council as traditionally bred, high-oleic soybeans. Although marketing of the genetically modified Plenish and Vistive Gold oils faced regulatory challenges in Europe and China, these were cleared in 2017 to permit global sales of these oils.

A challenge for these technologies, as with all novel crop traits, is the need to breed them into the many genetic backgrounds that have been optimized for production in the different climate zones and soil types in which soybeans are grown. With consumer opinion and now FDA regulation targeting partial hydrogenation as a means to reduce PUFA content of oils, this breeding bottleneck was (and still is) an ongoing barrier to efforts for recovering market share for soybeans in the U.S. and elsewhere. This barrier was recognized by the United Soybean Board (USB) in 2012 when they decided to provide funding to Dupont-Pioneer and Monsanto to accelerate the breeding of high-oleic cultivars to cover up to 80% of U.S. soybean acres by 2020, as well as funds for an advertising campaign to encourage farmers to begin growing the high-oleic varieties (United Soybean Board, 2012). Without this injection of funds (a planned total of $60 million over 3 years), industry projections predicted only 5%–10% coverage by 2020, at a cost to farmers of over $1 billion in lost crop sales (Wilson, 2012; United Soybean Board, 2013).

In many oilseed crop species, the FAD2 desaturases that produces PUFA in seeds are encoded by genes that are expressed throught the plant. For example, canola is an allotetraploid and contains four *FAD2* isogenes, designated *FAD2.A1, FAD2.A5, FAD2.C1*, and *FAD2.C5* (where the nomenclature indicates the subgenome (A or C) and the chromosome location of the gene). The *FAD2.A1* isoform contains deletion and insertion events that preclude it from encoding an active enzyme. The *FAD2.A5*, *FAD2.C5*, and *FAD2.C1* isoforms encode closely related proteins (>90% sequence identity) and mutations in these genes demonstrate that they each contribute to PUFA synthesis in all tissues of the plant. In the 1990s, several breeding programs produced lines with 70%–85% 18:1 (and 20% to 8% PUFA), presumeably by combining mutations at the three remaining loci. Most lines grew well in greenhouses, but when planted in field plots lines with the highest 18:1 levels failed to thrive. Arabidopsis *fad2-2* plants are damaged and killed by low temperatures (Miquel et al., 1993), suggesting that the canola lines with the lowest FAD2 expression are also susceptible to the low temperatures that are typical in the field during planting and early growth. To better understand these results, Bai et al. (2019) identified a series of hypomorphic and null mutations in the FAD2.A5 isoform and then combined four of these with null mutations in the other two isozymes, FAD2.C5 and FAD2.C1. The resulting mutant lines contained 71%–87% 18:1 in their seed oil (21% to 4% PUFA), compared with 62% in wild-type controls. All the mutant lines grew well in a greenhouse, but in field experiments a clear demarcation in plant performance was observed. Mutant lines containing less than 80% 18:1 in the seed oil were indistinguishable from wild-type controls in growth parameters and seed oil content. By contrast, lines with more than 80% 18:1 in the seed oil had significantly lower seedling establishment and vigor, delayed flowering and reduced plant height at maturity. These lines also had 7%–11% reductions in seed oil content. These results define the practical limit to increasing oil 18:1 content in canola (Figure 6), and by extension other crop species that rely on constitutively expressed *FAD2* genes for seed PUFA synthesis.

One strategy to lower oil PUFA while maintaining required PUFA synthesis in other tissues of the plant is to use seed-specific RNA-interference or artificial microRNA approaches. These have been applied to canola (Peng et al., 2010; Lee et al., 2016) and a number of other species, including cotton (*Gossypium hirsutum*; Liu et al., 2002), camelina (*Camelina sativa*; Nguyen et al., 2013), linseed (*Linum usitatissimum*; Chen et al., 2015), and safflower (*Carthamus tinctorius*; Wood et al., 2018). A caveat to these approaches is that investigations of the Arabidopsis *fad2-2* mutant indicate that normal seed development and germination both require some minimal content of PUFA, particularly at low temperatures (Miquel and Browse, 1994). Very strong knockdown of seed *FAD2* expression in oilseed species may likewise compromise seed physiology and crop viability.

## Conclusion

Vegetable oils make a large contribution to calorie intake in human diets, but the fatty acid compositions of oils from most oilseed crops are not ideal for human nutrition and the needs of the food industry. On one hand, saturated fatty acids are a known risk factor for coronary heart disease, obesity, and other diseases common in developed countries. On the other hand, polyunsaturated fatty acids can become rancid during storage and processing. Discoveries in lipid biochemistry and biotechnology over the last 30 years have provided the means to ameliorate both of these challenges. Genetic engineering of oilseeds has successfully reduced saturate content of oils, both by transgenic expression of fatty acid desaturases and by down-regulation of the acyl-ACP thioesterase responsible for 16:0 unloading from the plastid fatty acid synthase. The original, industrial-chemistry solution to the PUFA problem, initiated in the early 20th century, was to process oils by partial hydrogenation. This process leads to the production of *trans* fatty acids that are actually more damaging to human health than saturated fatty acids, with epidemiological estimates blaming them for half a million excess deaths worldwide each year during much of the 20th century. The dangers of foods containing *trans* fats were well established by 1998, but it has taken more than two decades for legislation in many countries to ban them from food. Indeed, more work is needed to stop production and consumption of *trans* fats in countries around the world. Ironically, identification and cloning of the *FAD2* gene that encodes the gateway enzyme of PUFA synthesis in 1994 provided the tools and understanding to engineer high-oleic, low PUFA lines of many oilseed crops, meaning that the solution to the *trans*-fat problem has been available for over 25 years.

## Author Contributions

All authors listed have made a substantial, direct, and intellectual contribution to the work and approved it for publication.

## Funding

Research on lipid metabolism in our laboratory has been funded by grants from the US National Science Foundation (grants MCB-0420199 and IOS-1339385), the USDA National Institute of Food and Agriculture (grants 2010-65115-20393 and 2018-67013-27459), BASF Innovation Center Gent, and the Agricultural Research Center at Washington State University.

## Conflict of Interest

The authors declare that the research was conducted in the absence of any commercial or financial relationships that could be construed as a potential conflict of interest.

## Publisher’s Note

All claims expressed in this article are solely those of the authors and do not necessarily represent those of their affiliated organizations, or those of the publisher, the editors and the reviewers. Any product that may be evaluated in this article, or claim that may be made by its manufacturer, is not guaranteed or endorsed by the publisher.
